# “Created Anew”: Notes on Black Queer Intersectional Joy

**DOI:** 10.1089/heq.2023.0132

**Published:** 2025-01-20

**Authors:** Marie-Fatima Hyacinthe, Elle Lett

**Affiliations:** ^1^ Department of Social and Behavioral Sciences, Yale School of Public Health, New Haven, Connecticut, USA.; ^2^ Certificate Program in Women’s, Gender, and Sexuality Studies, Yale Graduate School of Arts and Sciences, New Haven, Connecticut, USA.; ^3^ Children’s Hospital of Philadelphia, Philadelphia Pennsylvania, USA.; ^4^ Center for Anti-Racism and Community Health, University of Washington School of Public Health, Seattle, Washington, USA.; ^5^ Center for Applied Transgender Studies, Chicago, Illinois, USA.

**Keywords:** Black, queer, intersectionality, community-based research

## Abstract

While a focus on intersectional oppression elucidates important structural influences on health, it also obfuscates elements of how oppressed communities view themselves and their experiences. Black queer intersectional joy is one such element, and exploring this concept provides different openings for researchers to build solidarity with and produce relevant research alongside communities facing class oppression, heterosexism, racism, and cissexism. This article provides examples of Black queer intersectional joy, as well as potential problems and opportunities for engaging this concept.

## Introduction: Black Queer and Trans Joy in the Wake

Public health researchers take special care to frame research questions in ways that are relevant to lived experiences, utilizing concepts like “social determinants of health” to trace the impacts that societal structures have on health outcomes. Increasingly, researchers have paid special attention to communities facing “interlocking systems of oppression,” continuing the tradition of borrowing from other academic disciplines as they incorporate the theory of intersectionality from its original roots in Black feminist theory.^1–3^

Much of the work invoking intersectionality has used the theory as an analytical tool to identify the peculiarities and unique manifestations of oppression for individuals subject to multiple forms of social-structural marginalization. From these investigations, we see descriptive analyses of health inequities by cross-stratified social identities and the development of the nascent field of intersectional stigma.^4,5^ However, as the focus on stigma and sometimes resilience take center stage, we argue that these studies occlude the various ways that people facing these interlocking systems of oppression carve out spaces for joy and other life-sustaining practices.

In her 2016 book, Black studies scholar Christina Sharpe describes the current era as being specifically shaped by the Trans-Atlantic Slave trade, chattel slavery, and institutional racism. She uses the imagery of being “in the wake” of a ship to describe how global and local structures, as well as individual lives, are impacted by the ongoing ramifications of these centuries of violence and exploitation.^6^ Sharpe’s imagery might resonate for public health researchers focusing on the historical and ongoing causes of health inequities. However, just as important as Sharpe’s assertion that we are living “in the wake” is her conclusion that although oppressed people face various types of discrimination and violence, “we are not only known to ourselves and each other by that force.”^6^

This article attempts to explore joy as another force, which guides, motivates, and affirms the lives of Black queer and trans people. We argue that joy is as important for public health researchers to consider as structural oppression is. From our positionality as public health researchers and Black queer women, we offer that endeavoring to recognize the joy that communities cultivate in the midst of oppression is equally important for researchers who wish to be in solidarity against oppression. In our discussion of Black trans and queer joy, we do not mean to flatten or simplify joy. We recognize that today’s hyper-individualistic and capitalistic society believes that joy is equivalent to purchasing power. However, we look to Black queer feminists such as Audre Lorde, who describes “places of possibility within us.”^7^ Joy, which may or may not be related to external circumstances, is one such place of possibility. It can be a foundation or motivation for resistance, as much as it can be a place of respite in an unyielding world.

Black studies scholar Saidiya Hartman provides an analysis of the role of practices such as song and dance for enslaved communities: “Rather than think about these practices as providing a reprieve from domination, we must think about pleasure not only in the context of domination but also as an articulation of these tensions, limits, fissures, wounds, and ravages.”^8^ In keeping with this, we argue that Black trans and queer joy can be a useful analytic for public health, as it can illustrate the points where systematic oppression is navigated, mitigated, or subverted. Furthermore, an understanding of joy demands that public health scholars deal with Black trans and queer communities as groups of people with lived experiences and informed analyses, rather than abstract examples of the effects of structural oppression. It is from this conviction that we take the phrasing, “created anew.” We argue that, in comparison to the relationship between harm and resilience that is the primary focus of intersectional discourse in public health, unique expressions of joy are what is freshly generated by existing at a particular intersectional position—not in response to harm, but perhaps despite it, or even completely independent from it. Respecting the role of Black queer joy in individual and community experiences can help to facilitate these possibilities that Lorde describes, and pushes back against logics of deficiency, which often accompany data about the health inequities that Black trans and queer people face.

Drawing on a wide range of theories that come from and are utilized by communities facing intersectional oppression, we propose some considerations for thinking about Black joy in public health spaces. Specifically, we propose that recognizing Black queer joy in its many manifestations redirects researchers to focus on communities not as collections of deficits or poor health outcomes, but rather as made of people with desires, hope, and joy.

## An Example

I (E.L.) want to provide an anecdote that captures an instance of “intersectional joy.” As a Black, transgender woman, navigating a sociopolitical environment that is categorized by systemic racism, misogyny, and cissexism, coalescing into transmisogynoir^9,10^ can be destructive and painful. Much work has been expended to capture individual responses to this prolonged trauma, including work on how individuals respond to structural harms through resilience.^11^ However, little scholarly work in public health reflects on what is created anew; what manifestations of strength and power exist for people at intersectional social positions. This work would not be an emphasis on our trauma responses but a light shone on how we manifest health and joy against the backdrop of a fundamentally inhospitable world.

I was attending the public launch of a collaborator’s research project as an invited discussant. In this role, I was on a stage as a panelist and I purposely showed up in my full trans womanness; big, broad-shouldered and Black, in a black and red flower-embroidered dress I had made, and boots that matched perfectly. As a trans woman who tries to reject the idea of “passing,” I purposely choose a “loud” presentation to exemplify gender expression outside of a cisnormative standard. After the panel, a prominent Black woman public health scholar approached me and asked if she could “speak to me like a church mother.” For context, church mothers, in the Black southern incarnation of Christianity that I grew up in, are informal positions of honor held by elderly women in the church who function as spiritual guides, mentors, and confidantes for younger women. Initially, I was hesitant but invited her to proceed. She first complimented me on my dress and told me that I looked beautiful and presented well. Then she gently gave feedback on how I sat on stage. My dress was just above the knee and she identified moments where I had a few near misses as my dress moved as I shifted in my seat. She assured me that I had not embarrassed myself but gave a few pointers on how to sit, and even demonstrated a few positions.

The reader, potentially an outsider, not familiar with the nuances of Black Christian culture may interpret this behavior as “chastising” or policing a (trans)woman’s presentation in service of “professionalism,” reinforcing standards that implicitly hypersexualize and stigmatize women’s bodies and uphold patriarchal dominance. But, I invite the reader into a different experience: this moment happened within a special bubble of Black Christianity, against the backdrop of the “church hurt” or rejection that I and many Black queer and trans individuals experience from their faith communities and families.^12^ In this context, this older, Black, cisgender woman, had treated me like she would a young girl at church, serving as a mentor and guide. In that moment, through the briefest of exchanges, she had inducted me into Black womanhood, over the boundaries of faith and prejudice that had previously kept me out. There was no pretense about me having to establish or defend my gender; it was assumed, validated, and accepted.^1^

The joy I felt in an otherwise mundane brief exchange is something that only I as a Black trans woman, who has navigated the very specific system of transmisogynoir in the United States, could have experienced. This is one of the experiences of girlhood that up until that point I had always imagined, and was for the first time experiencing. There is another discussion to be had about respectability in presentation, and to what degree we have to consider other people’s experiences of our body in how we present. Regardless, all of that complexity is wrapped into navigating the world as a girl and woman and this stranger had invited me in to share that. Most importantly, it was not a moment where I had to be resilient or resistant; instead, it was a moment where I could be soft, a brief moment to heal the inner girl who had previously been harmed, and experience Black gender euphoria.^13^ This is what we mean when we say intersectional joy: in the same way that Black feminism teaches us to value unique experiential knowledge,^2^ we have to recognize the healing potential of experiences of joy that can only be felt by those who exist at specific intersections. This anecdote is one example of experiences I have accumulated over a lifetime that contribute to my healing and health.

## Rethinking Risk, Joy, and Community in Public Health Research

As the example above shows, Black queer and trans joy is complex, varied, and sometimes fraught. Consider the example of O’Shae Sibley, who was killed in 2023 while voguing near his car at a gas station in Brooklyn, New York.^14^ Even in a city as putatively welcoming as New York, Sibley’s example of Black queer joy was still met with fatal violence. Black trans feminist author and activist Raquel Willis recognizes this sort of interpersonal violence as a manifestation of structural oppression: “As a Black trans woman, my existence was a protest against white supremacy and the cisheteropatriarchy.”^15^ To exhibit joy publicly while being Black, queer, and trans is to reckon with a daily ongoing risk of varying types of violence—from the structural to the intimate.

Public health has a long history of “managing risk,” which often includes ways to augment behaviors so that they carry “less risk.” It quickly becomes clear that these types of public health risk interventions cannot and will not help in situations that Willis describes: part of this legacy is the way that Black trans and queer behavior has been pathologized and positioned as a motivator for “risk behaviors.” Activist and writer Kenyon Farrow describes how dance club spaces that cater to Black queer and trans people have often been positioned as spaces of illicit drug use and sex, leading to policing and criminalization of these spaces and obscuring the role these spaces of joy provided support during the first two decades of the HIV epidemic.^16^ This framing continues in the ways that certain activities and communities are deemed inherently risky or problematic in public health rhetoric.

If one goal of public health research is to contribute scholarship that helps to dismantle intersecting systems of oppression, paying attention to how people facing these oppressions foster joy can offer more opportunities for the type of research that can undergird real solidarity between researchers and the communities they work with. Farrow goes on to describe how house music spaces hosted fundraisers for HIV-related causes and challenged racism and gender norms in the nightlife space.^16^ They provided a space to commune, which in turn led to care networks. Some elements of this can be seen in queer and trans nightlife spaces today, as party hosts post community norms on social media that highlight harm reduction services for drug use and non-carceral approaches to safety as participants travel to and from the party. These are historical and current examples of public health questions being addressed through Black trans and queer joy; if public health researchers take these examples seriously, the field can lend itself to supporting and promoting these kinds of actions.

Thinking seriously about joy and different approaches to risk should force public health researchers to reconsider how we use the word “community” itself. Black feminist scholar bell hooks uses a definition that describes a community as a “group of people who have developed some significant commitment to rejoice together, mourn together, delight in each other, and make each other’s conditions our own.”^17^ This definition is different from public health’s, where we use the community as a more acceptable way of saying “population,” or “research participants.” Public health focuses on how these groups are connected by poor health outcomes or exposure to the forces of structural oppression, but if we recognize that communities are cohered by joy, as well as by shared vulnerability, we can better recognize the agency and goals of these groups. Intersectional joy then, is not an object of scrutiny, but rather an element of people’s lived experiences that can shape our work.

## Reproductive Justice: Applying Intersectional Joy to a Public Health Research Agenda

As the anecdote above asserts, intersectional joy can promote healing and health. Writing about reproductive justice (RJ), Black feminist Science and Technology Studies scholar Ruha Benjamin describes “the full range of life-affirming practices” that communities facing racial oppression utilize.^18^ While much of the visible work of RJ is resisting and responding to medical racism, sexism, ableism, classism, heteronormativity, RJ provides more than a corrective to current politics as they manifest in abortion bans and other legislation. Rather, it is a visionary, transformative framework that works toward creating a world where people can choose to parent or not in dignity and have the necessary resources to actualize those choices.^19^ In fostering “life-affirming practices,” RJ contends with joy, risk, community, and dignity.

Understanding this approach mandates that researchers aligned with the RJ movement understand abortion bans, criminalization of gender-affirming care, and bans on teaching histories of racism and oppression as interconnected threats. This analysis operationalizes the tenets of intersectionality in terms of focusing on those who are multiply-marginalized and affected by co-constitutive forms of oppression. Yet, it also provides room to consider the solidarities and coalitions that are formed not only in response to this oppression, but from a desire for a radically different reality. RJ provides the opportunity to look at how people envision raising their children, including trans children, gender nonconforming children, and Black children, “in dignity,” and to produce research that aligns with those desires in the face of these oppressive legislative moves.^20^ Engaging RJ with an understanding of intersectional joy might produce research that engages and elucidates “places of possibility” among these compounded oppressions.

## An Invitation to Start Anew

Public health researchers interested in Black queer joy should be aware of the potential issues in this work. Since so much of Black queer life is already under the proverbial microscope, researchers run the risk of further fetishizing Black queer joy. In “Black Sex in the Quiet,” Shoniqua Roach describes the ways that forcing Black women’s sexuality to “speak for” all Black women exacerbates the fungibility of Black women, rather than giving rise to more nuanced conversations.^21^ We similarly caution against projects that presume that making Black queer joy more “visible” will inherently mitigate oppression for Black trans and queer people. Rather, we encourage scholarship that is attentive to the myriad ways that Black queer joy surfaces and that is committed to actualizing the moments of possibility that come out of that joy.

Additionally, we are wary of projects that would position joy as synonymous with “resilience.” As has been demonstrated elsewhere, resilience frameworks focus on individuals responding to and adapting to structural harm.^11^ Our understanding of Black queer joy is not focused on the individual level, but rather at the community level. Furthermore, we are not interested in how joy might help Black queer and trans people adapt to harm, but rather how it can provide space to imagine a world without these structural harms.

So, with these critiques and concerns laid out, what direction are we providing? We are most certainly leaving you all with more questions than answers. That is because joy has so rarely been considered for people who exist at our overlapping and distinct intersections. It is hard to imagine a study that measures Black queer joy when the scientific record has so long ignored and pathologized it. Instead, what we leave you with is perhaps an inkling of that feeling of intersectional joy; that moment when a Black woman can be “soft” and heal—we invite you to chase it; to try to create conditions that nurture it with your participants and co-collaborators, with your ideas, and with a radical re-envisioning of what health can be.

## Notes

^1^
Importantly, the “joy” of this experience may only be legible by degree based on proximity to the experience of the author (E.L.). Those individuals with more identity overlap will be able to connect with the anecdote more easily by analogy to their own lived experience, while those with greater distance across more axes of identity (and power relations) will have less access to it and will only be capable of a partial appreciation. We as authors experience this in our own interpretation of the anecdote as Black queer women but of cis and trans experience not reared in the same southern Christian contexts. A similar experience can be said of Beyoncé’s pivotal “Renaissance” album, and its celebration of Black queer house music, ballroom, and culture–appreciated by fans generally, but uniquely cherished by the Black queer consumers who seek to live the joy echoed in the album throughout their daily lives (Crumpton, 2022).

## Abbreviations Used

HIV: Human immunodeficiency virus
RJ: Reproductive justice
